# The Influence of Entrepreneurs’ Psychological Capital on Their Deviant Innovation Behavior

**DOI:** 10.3389/fpsyg.2020.01606

**Published:** 2020-08-28

**Authors:** Wenhai Xu, Shouzheng Zhao

**Affiliations:** ^1^School of Law, Tongji University, Shanghai, China; ^2^School of Law, Shanghai International Studies University, Shanghai, China

**Keywords:** entrepreneur psychological capital, deviant innovation behavior, questionnaire survey, relevance, analytical hierarchy process

## Abstract

This study explores the influence of psychological capital on the deviant innovation behavior, with the purpose of realizing the application and development of positive psychology in the field of innovation and creation. First, the data was obtained based on the questionnaire, and the Likert scale was adopted to measure the variables effectively, in which 1 point means “never,” 2 points mean “rarely,” and 5 points mean “always.” Second, the SPSS 26.0 statistical analysis software was adopted, and a statistical analysis was made on the correlation among deviant innovation, psychological capital, work values, and work remodeling. Third, the relationship between psychological capital and deviant innovation behavior was explored using the analytic hierarchy process (AHP). The results showed that the reliability of each scale is good, Cronbach’s α coefficients are all higher than 0.8, and the fitting effect of the four-factor model is the best, proving that the highest differentiation validity can be achieved using the proposed method. Furthermore, there are significant correlations among entrepreneur’s psychological capital, entrepreneur’s work values, and entrepreneurship work remodeling and deviant innovation behavior, among which the psychological capital and work values are the most correlated with deviant innovation. With the psychological capital of entrepreneurs as the adjustment variable and the interaction added, the explanation rate of the level equation is increased from 17 to 24.2%. Therefore, the psychological capital of entrepreneurs plays a very big role in regulating work values and deviant innovation behavior. In the current environment of innovation and entrepreneurship development, it is necessary for entrepreneurs to give full reign to the regulatory role of their own psychological capital, so as to promote the development of self-active deviant innovation activities and encourage employees to actively innovate and create.

## Introduction

Under the current social and economic development, the mass entrepreneurship and innovation program has been in full swing in China. Entrepreneurship and innovation are key influencing factors for driving national economic growth as well as being essential factors for motivating enterprises to participate in international competition (Kim and Ryu, 2017; Patel et al., 2018). As the entrepreneurship field develops, the development of the innovation field and the change in management modes of enterprises, face severe challenges. The innovation behavior of employees is the key to improving the innovation capacity of enterprises (Sorescu, 2017). Under the influence of such an environment, enterprises have made great changes in their attitude toward employees’ innovative behavior, which to a certain extent promotes the development of employees’ innovative behavior (Tandon et al., 2017; Vedin et al., 2017). However, due to the impact of objective factors such as innovation resources and business risks, it is still difficult to recognize and carry out some creativity activities in the enterprise organization, so, deviant innovation has become more common in this background. As a result, increased attention has been given to the exploration of deviant innovation mechanisms and influencing factors. At present, research on employees’ deviant innovation behavior focus more on its destructive aspect (Shaw et al., 2016). Some scholars suggest that deviant behavior, based on promoting the healthy development of the organization, could significantly improve performance (Shoaib and Baruch, 2019). Nevertheless, research has shown that inclusive leadership was conducive to promoting employees’ deviant behavior (Pandey et al., 2017), and psychological ownership played an intermediary role between leaders and employees, affecting deviant behavior (Malizia, 2018). It has been found that most studies on deviant innovation behavior focuses on employees. Furthermore, psychological capital is developed based on positive psychology. It was originally proposed based on the understanding of the personality characteristics that affect the efficiency of an individual’s work. Then, (Luthans et al., 2010) attributed psychological capital to a positive psychological state, including self-efficacy, hope, optimism, and resilience in positive psychology, and the investment and development behavior could promote the individual competitive advantage. As a result, the concept of psychological capital can be further improved (Yun and Kang, 2018; Clausen et al., 2019; Verhiel et al., 2019). Employees with an optimistic attitude tend to have a higher sense of self-efficacy, so they have a more positive work value orientation, which affects their deviant behavior psychologically (Tharek et al., 2018). At present, there are very few studies on introducing psychological capital into deviant innovation behavior.

Accordingly, a special group of entrepreneurs was selected as the research objects in this study, and the concept of psychological capital was introduced. Based on the questionnaire survey and statistical analysis, this study introduced two elements that were closely related to psychological capital – work values and work remodeling – so as to examine the role of psychological capital of entrepreneurs in their deviant innovation behavior. The purpose of this study is to provide some reference for the relationship between psychological capital and deviant innovation behavior, based on positive psychology.

## Literature Review

### Research Status of Deviant Behavior

In view of deviant behavior, scholars in related fields have carried out corresponding research work. Fassauer (2018) defined innovation behavior as “expected deviant,” and analyzed and discussed the impact of enterprise management control on employee innovation behavior (Fassauer, 2018); Fried (2017) explored the impact of management control on deviant behavior in the context of innovation (Fried, 2017); Kyegombe et al. (2017) used a special analysis, continuous comparison, and relevant cases of deviant behavior to explore the mitigation of teacher-student relationships (Kyegombe et al., 2017); Michel and Hargis (2016), based on the theory of psychological needs and self-determination, discussed the relationship between deviant behavior in the workplace and organizational injustice, and found that it was very important to cultivate employees’ intrinsic behavior motivation in the workplace (Michel and Hargis, 2016); Shoaib and Baruch (2019) introduced the concept of deviant behavior into the moderate intermediary framework of incentive and organizational justice perception. Based on the causal model and structural equation model analysis, the results showed that incentive for employees has direct, indirect, or conditionally indirect effects in their deviant behavior (Shoaib and Baruch, 2019).

### Research Status of Psychological Capital

Zhang et al. (2017), based on social cognition and social exchange theory, found that customer psychological capital had a greater impact on knowledge sharing behavior in the virtual innovation community (Zhang et al., 2017); Bakker et al. (2017) discussed the influence of psychological capital on depression symptoms of veterinary students, and concluded that the components of psychological capital could be learned and strengthened through relevant interventions (Bakker et al., 2017); Yim et al. (2017) discussed and analyzed the mediating role of psychological capital in occupational stress and turnover intention of nurses, and the results indicated that psychological capital had some mediating role in the stress and turnover intention of nurses (Yim et al., 2017); Cuadra-Peralta et al. (2018) explored the relationship between psychological capital and the expected attitude of employees in state-owned and private enterprises, and found that there was a significant positive correlation between psychological capital and expected attitude, as well as a significant correlation between psychological capital and job satisfaction (Cuadra-Peralta et al., 2018); Yang and Yang (2019), according to the investigation and analysis of the employees of small and medium-sized venture capital companies in South Korea, found that the actual leadership of the CEO could significantly improve the positive psychological capital of employees of small and medium-sized enterprises (Yang and Yang, 2019).

In summary, the above findings show that the current research work on deviant behavior is mostly focused on its destructive aspects. Research on deviant innovative behavior is mostly directed at employees and there are few relevant research findings. Furthermore, it can be found that psychological capital has a greater impact on the working state and work behavior of employees. At present, however, there are few studies on introducing psychological capital into deviant innovation behavior, so it is necessary to combine them (Chen, 2018; Shen et al., 2019).

## Materials and Methods

### Theoretical Basis of Deviant Innovation Behavior

The so-called deviant innovation refers to the behavior of an employee who continues to improve or implement an idea proposed after it is denied by the superior supervisor. This kind of innovation behavior is generally proposed by employees in the interests of the organization, while the organization refuses the innovation of employees due to the inconsistency between the corresponding innovation scheme and the overall strategic development or the consideration of risk minimization, so the employees adhere to and carry out the behavior in private. In the concept of deviant innovation, “innovation” and “deviant” are two major elements. The specific expression of the innovation element is a creative process in which employees have new ideas and then promote the implementation of ideas. Although new products are not necessarily generated in this process, it is considered to be an act of good will because it is based on improving new ideas (Ding et al., 2018). Deviant elements are presented in violation of the expectations of norms in the social environment. Under the current development situation, the research on deviant innovation is still in its initial stages, and the deviant innovation behavior of employees is private and risky. From the perspective of enterprise employees, the original order and rules of the enterprise organization have an impact on their deviant innovation behavior. Furthermore, the results of deviant innovation behavior of employees are affected by their position, work values, psychological authorization, and other factors. At present, research mainly focuses on employees’ deviant innovation behavior. However, unlike ordinary employees, an excellent entrepreneur shall have a keen perception, strong decision-making ability, and good risk control ability, which in fact are more likely to produce deviant innovation behavior.

**FIGURE 1:**
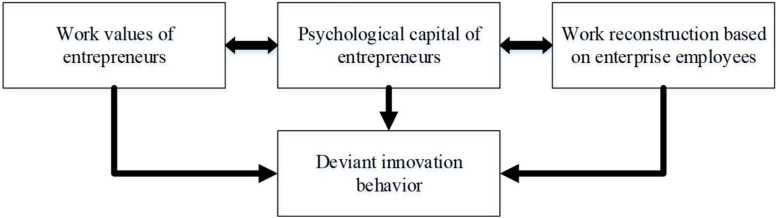
Variable selection and the establishment of conceptual research model.

Corresponding to the development stage of deviant innovation, the measurement of the relevant scale of deviant innovation is still relatively inadequate, and only a few scholars have studied it, such as the deviant innovation scale developed by Lin et al. (2018) that contains eight topics, and the deviant innovation scale developed by Alexy et al. (2016) that contains five topics. This innovative measurement scheme has been proven to have good reliability and validity. In research on the influence of entrepreneurs’ deviant innovation behavior, the above two deviant innovation scales were synthesized and simplified appropriately. The corresponding variables were selected, and the conceptual research model was established (Figure 1).

### Psychological Capital Based on Positive Psychology

Positive psychology began to develop in the late 20th century. At present, it has achieved good results in the fields of health, personality, counseling, and other branches of psychology. It is a field of research based on the relationship between the positive quality and mentality, positive emotion, potential development, and happiness promotion of the corresponding individual. Based on this, (Luthans et al., 2010) put forward the concept of positive psychological capital, which could also be called psychological capital (Friend et al., 2016; Gander et al., 2018). Luthans et al. (2010) believed that psychological capital was mainly composed of four structures: self-efficacy, resilience, optimism and hope, which were conceptually independent and had differentiated validity on the basis of empirical evidence. Regarding the definition of the attribute of psychological capital, the state theory, trait theory, and integration theory are the three main viewpoints (Clausen et al., 2019).

Since the concept of positive psychological capital was put forward, it has been favored by psychologists and management scientists. Currently, the measurement questionnaire of positive psychological capital research mainly consists of one dimension, three-dimension, four-dimension, and multi-dimension theories (Kyösti et al., 2019). Table 1 shows the corresponding structure and scale of positive psychological capital.

**TABLE 1:** Structure and scale of positive psychological capital.

**Specific dimensions**	**Composition of structural elements**	**Corresponding scale**
One dimension	Self-esteem	Mental capital scale
Three dimensions	Hope, optimism and toughness	Mental capital scale
	Self-efficacy, optimism and toughness	Mental capital status scale
Four dimensions	Hope, optimism, self-efficacy, and toughness	Psychological capital evaluation scale
	Hope, optimism, toughness, and self-efficacy	Mental capital status scale
	Self-esteem, self-efficacy, and emotional stability, control point	Core self-evaluation construct scale
	Hope, optimism, self-efficacy, and toughness	Positive psychological capital questionnaire
	Realistic hope, optimism, self-efficacy, and toughness	Psychological capital questionnaire
Multiple dimensions	Emotional stability, openness, extraversion, responsibility, and agreeableness	Psychological capital questionnaire
	Hope, optimism, self-efficacy, toughness, and sincerity	Positive psychological capital evaluation scale
	Modest and steady, tolerance forgiveness, confident and brave, tenacious and stubborn	Scale of psychological capital in China

### Work Values of Entrepreneurs and Deviant Innovation

**FIGURE 2:**
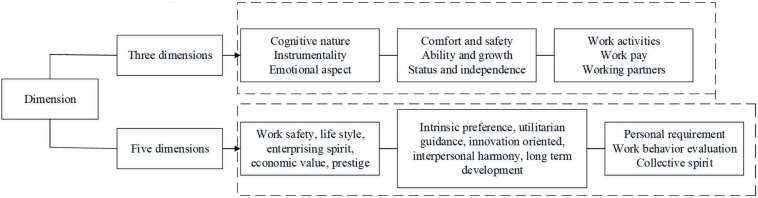
The scale of entrepreneur’s work values and its corresponding dimensions.

Work value refers to a kind of preference and attitude toward the work, which will exert an impact on the degree of investment and innovation behavior of entrepreneurs in the workplace (Armstrong et al., 2018; Antonio et al., 2020). Because of the differences in management difficulties, the work values of entrepreneurs are different. For entrepreneurs who focus more on utility orientation and material interests, work efficiency improvement, and self-interest maximization in their work, the success of their deviant innovation behavior is closely related to high return. In this case, the efficiency between input and output of deviant innovation and the satisfaction of demand for maximizing personal interests are at a high level. For entrepreneurs who emphasize internal preferences, the matching of interests and needs in their work is vital. As a result, for entrepreneurs who are more sensitive in the perception of work value and significance, the deviant innovation is the realization of their own work value, the value embodiment of work preference, and the pursuit of company interests (Chen, 2019; Shen and Ho, 2020). Moreover, for entrepreneurs who focus on interpersonal harmony and a harmonious working atmosphere in their work, and those who expect equal communication between superiors and subordinates, will be more inclined to innovation privacy in the embodiment of deviant innovation behavior. For entrepreneurs who emphasize creativity and freshness in their work, regard new things and knowledge more highly, and have outstanding creativity and imagination, deviant innovation behavior is actually the maintenance of innovation projects and creativity. While for entrepreneurs who are concerned about long-term development in their work and those who hope to gain social resources through continuous accumulation, the manifestation of their deviant innovation behavior is actually the process of individual experience accumulation of innovation and the behavior process of insisting on self-innovation belief. At this time, the high return characteristics of deviant innovation promote the possibility of further improvement and development of entrepreneurs (Armando et al., 2020). Based on the above statements, for entrepreneurs, long-term development, driven by work values has the greatest positive impact on deviant innovation. Therefore, the work values of entrepreneurs are introduced as the intermediate variables to explore and study the relationship between entrepreneurs’ psychological capital and deviant innovation behavior. Figure 2 shows the scale of entrepreneur work values and its corresponding dimensions (Zheng and Liu, 2020).

### Work Remodeling and Deviant Innovation

In fact, work remodeling refers to an active way of behavior change, which is derived from the activities of the perspective of individual employees and the bottom-up work design. Its idea is to express all the defects, concepts, or relationship changes in the work of enterprise employees. On the basis of their own and enterprise needs, the employees of an enterprise will positively affect the organization by redesigning the working methods, and achieve the goal of balancing the working resources and requirements by changing the individual working ability and working demand.

According to relevant research, the motivation of employees’ work remodeling comes from the need for work control, self-image, and interpersonal relationships. Among them, the need for work control is related to tasks. The relationship-related work remodeling has an impact on the level of communication between employees and other people. Whether it is work remodeling related to work tasks or relationship, it is the need for employees to build a positive self-image, improve their personal ability, and enhance their sense of work value with the team, life, or organization. Work remodeling is closely related to a positive mentality and personal ability improvement, which is helpful for establishing positive psychological attitudes and promoting innovation behavior. Similarly, whether the work remodeling behavior of entrepreneurs will promote the occurrence of their deviant innovation behavior is also helpful to explore the relationship between psychological capital and deviant innovation behavior. Therefore, the concept of entrepreneurial work remodeling was introduced, and the correlation between it and the research object was analyzed, thereby laying a foundation for the in-depth study of psychological capital and deviant innovation behavior (Li et al., 2019; Wu et al., 2019).

### Study Design

(1) Research Methods and Sample Selection

For research on the influence of entrepreneurs’ psychological capital on themselves and employees’ deviant innovation behavior, in this study, some entrepreneurs of high-tech and mature enterprises were selected as the research object. The questionnaire survey was used to obtain data. Combined with the offline questionnaire and online software survey, the way closer to entrepreneurs was utilized to achieve the distribution of the offline questionnaire. Considering the sensitivity and particularity of deviant innovation, the privacy of the questionnaire in this survey and research was protected, in order to ensure that individual entrepreneurs report their deviant innovation level in a truthful manner. Moreover, the data collection was completed anonymously, and it was promised that the data collection was only used for academic research purposes. To improve the effectiveness of the questionnaire, the offline questionnaire was distributed face-to-face, and the online questionnaire was mainly distributed in the form of “wenjuanxing” (SCM), a professional platform surveys. Among them, the online electronic questionnaire was mainly distributed in Hangzhou, Nanjing, Nanchang, Shenzhen, Shanghai, and other cities where high-tech enterprises are present, and the analysis and statistics of the recovered questionnaire were completed according to SCM.

(2) Research Tools and Data Analysis Methods

To ensure the effective measurement of variables, a mature and reliable Likert scale was selected to achieve the measurement, in which 1 means “strongly disagree” and 5 means “strongly agree.” For deviant innovation behavior, the Criscuolo deviant innovation scale was chosen, which included 5 topic options, such as “I will take the initiative to spend some time on research projects to enrich the project development.” For the work values of entrepreneurs, the Grounded Theory with good reliability and validity was used to build a five-factor work values scale, which included 20 topic options, such as “I hope the company can further improve the level of innovation and achieve long-term development.” For the measurement of psychological capital, the four-dimensional theory was chosen according to the above statement. For the measurement of entrepreneurship work remodeling, the highly reliable *The Development and Test of Work Remodeling Scale* published by Tims and Bakker was used, which included 21 item options. The scores were given according to the 5-point Likert method: 1 point means “never,” 2 points “seldom,” and 5 points “always.” The higher the corresponding score is, the more obvious the work remodeling behavior is.

**FIGURE 3:**
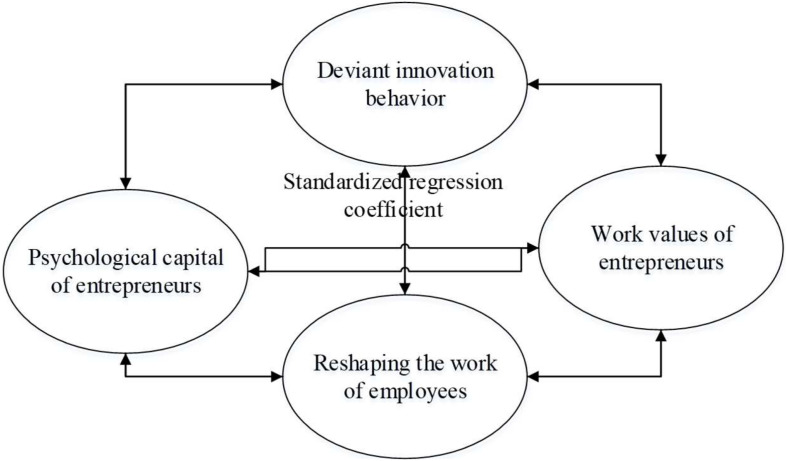
The establishment of the structural equation model for each selected variable. The results of model fitting.

To analyze and process the data, this study adopted the SPSS software, a method widely used in reliability and validity measurement and multiple linear analyses. Furthermore, the software was also applied to test the correlation among various corresponding variables, such as entrepreneur’s psychological capital, work values, deviant innovation behavior, and entrepreneurship work remodeling. This proves that psychological capital plays an important role in the adjustment of entrepreneur’s work values, employee’s work remodeling, and their deviant innovation behavior, by using an analytic hierarchy process (AHP). Among them, the calculation of Cronbach’s α coefficient based on reliability test is shown in equation 1, and the calculation of standard deviation is shown in equation 2. For the validity test, the confirmatory factor analysis method in the structural equation model was used to analyze the fitting effect of the model through the corresponding numerical value of the test index. The test indexes used for model fitting are chi square χ^2^, degree of freedom df, comparison fit index CFI, incremental fit index IFI, Tucker-Lewis index TLI, root mean square residual RMR, and root mean square of approximate error RMSEA. Figure 3 shows the structural equation model of each selected variable established.

(1) $$\alpha =\frac{\text{nr}}{\left[\left(n-1\right)\,r+1\right]}$$

where, n represents the number of questions in the scale measurement, and r is the average correlation coefficient between questions.

(2) $$\mathrm{SD}=\sqrt{\frac{\left[{\left(x_{1}-x\right)}^{2}+\ldots \,{\left(x_{n}-x\right)}^{2}\right]}{n-1}}$$

where, n suggests the number of research samples, and x_1_…x_n_ represents the corresponding value of each research sample.

## Results

### Questionnaire Results

The demographic characteristics of the samples were selected, as shown in Table 2.

**TABLE 2:** Demographic characteristics of research samples.

**Demographic variables**	**Category**	**Proportion**
Gender	Male	55.98%
	Female	44.02%
Age	26–30 years old	25.49%
	31–40 years old	27.30%
	41–50 years old	40.10%
	Over 50 years old	7.11%
Educational	Master’s degree or above	11.79%
background	Undergraduate	44.77%
	Junior college	20.56%
	High school or technical secondary school	14.42%
	Junior high school and below	8.46%
Years of working	10 years and above	6.9%
	6–10 years	43.5%
	3–5 years	29.4%
	1–3 years	20.2%
Nature of enterprise	State-owned enterprise	22.1%
	Private enterprise	62.3%
	Foreign enterprise	2.3%
	Sino-foreign joint venture	1.4%
	Others	11.9%
Industry	Manufacturing industry	13.8%
	Internet	3.7%
	Medicine	3.2%
	Real estate	12.1%
	Service industry	23.1%
	Consultation	4.3%
	Education	16.4%
	Others	23.4%

A total of 400 questionnaires were distributed, and 380 questionnaires were recovered. To further ensure the validity of the questionnaire, invalid questionnaires were eliminated from the recovered questionnaires. The corresponding screening criteria for an effective questionnaire is that the options of the corresponding questions are relatively real, and there are few consistent situations. Thus, there are 340 effective questionnaires based on this study, with a recovery rate of 95% and an effective rate of 89%. The demographic characteristics of the corresponding study samples are shown in Table 2. In general, the sample selection in this study covers different genders, ages, families, and education levels, which lays a good foundation for the universality and scientific feature of the follow-up study.

### Reliability and Validity Test of Each Variable Scale

With the purpose of testing and analyzing the measurement reliability and validity of the variables of entrepreneur’s psychological capital, entrepreneur’s work values, entrepreneurship work remodeling, and deviant innovation behavior, the analysis software SPSS 21.0 was selected and combined with the confirmatory factor analysis method to analyze the reliability statistics of the selected variables. Table 3 shows the reliability statistical analysis of each measurement.

**TABLE 3:** Reliability statistical results of each measurement.

**Measurement variables**	**Deviant innovation**	**Work values of entrepreneurs**	**Psychological capital of entrepreneurs**	**Reshaping the work of employees**
Cronbach’s α coefficient	0.884	0.959	0.942	0.885
Item number	5	20	4	21

The data in Table 3 shows that the Cronbach’s α coefficient values of the scale selected are all above 0.8, and the factor load and interpretation rate of cumulative variance of each measurement item corresponding to the scale are within the specified range. Hence, it was concluded that the scale selected is appropriate, and each scale measurement shows good reliability.

**FIGURE 4:**
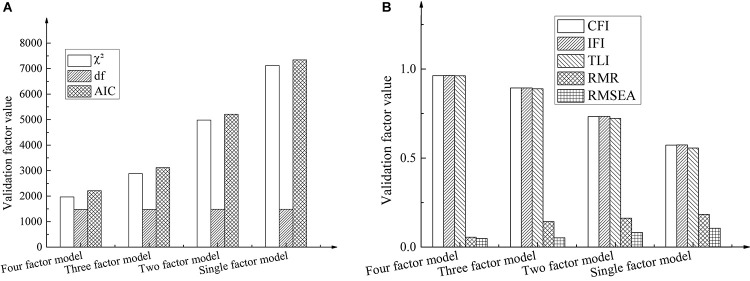
Model fitting results based on confirmatory factor analysis. **(A)** The results based on confirmatory factors c2, df, and AIC. **(B)** The results based on confirmatory factors CFI, IFI, TLI, RMR and RMSEA.

Based on the confirmatory factor analysis, the model fitting effect among the entrepreneur’s work values, entrepreneur’s psychological capital, entrepreneurship work remodeling, and deviant innovation behavior was analyzed. The corresponding data distribution and representation are shown in Figures 4A,B.

In Figure 4, the four-factor model represents deviant innovation, entrepreneur’s psychological capital, entrepreneur’s work values, and entrepreneurship work remodeling; the three-factor model represents deviant innovation + entrepreneur’s psychological capital, entrepreneur’s work values, and entrepreneurship work remodeling; the two-factor model represents deviant innovation + entrepreneur’s psychological capital + entrepreneur’s work values, and entrepreneurship work remodeling; the single-factor model indicates deviant innovation + entrepreneur psychological capital + entrepreneur work values + entrepreneurship work remodeling. Figure 4 suggests that the four-factor model has the best fitting effect. The specific performance of each confirmatory factor is χ^2^ = 1969.012, degree of freedom df = 1475, comparative fitting index CFI = 0.963, incremental fitting index IFI = 0.964, Tucker-Lewis index TLI = 0.962, root mean square residual RMR = 0.055, and root mean square error approximation RMSEA = 0.049. This shows that the four-factor model has the best discrimination validity.

### Correlation Analysis of the Selected Variables

**FIGURE 5:**
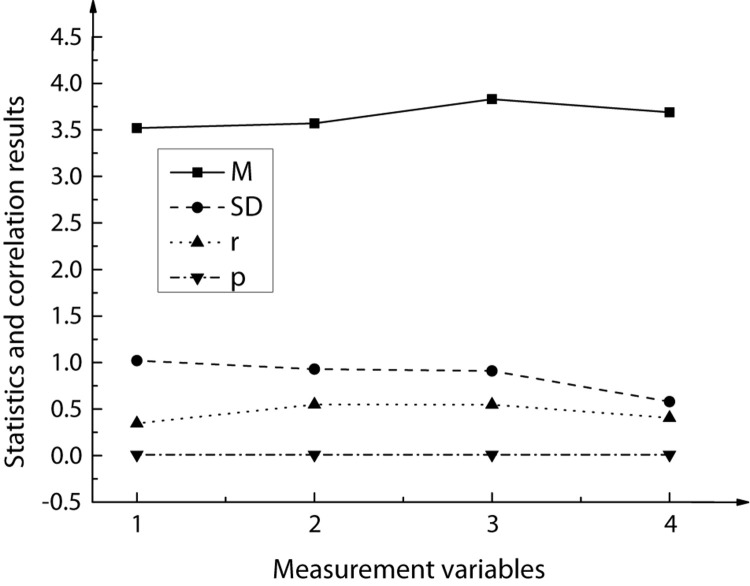
Descriptive statistics and correlation analysis results of variables (the variables represented by 1–4 include deviant innovation, entrepreneur psychological capital, entrepreneur work values, and entrepreneurship work remodeling).

With the purpose of analyzing and verifying the correlation among deviant innovation, entrepreneur psychological capital, entrepreneur work values, and entrepreneurship work remodeling, the mean m and standard deviation SD were selected for description and statistical analysis. Figure 5 shows the correlation analysis results of each selected variable.

The analysis of the data changes suggests that there is a significant positive correlation between entrepreneurs’ psychological capital and deviant innovation behavior. Specifically, the analysis coefficient is *r* = 0.549, and the significance level is *p* < 0.01. There is a significant positive correlation between entrepreneurs’ work values and deviant innovation behavior, specifically, *r* = 0.548, *p* < 0.01. Furthermore, there is a significant positive correlation between entrepreneurship work remodeling and deviant innovation, *r* = 0.406, *p* < 0.01. Generally speaking, the psychological capital and work values of entrepreneurs are the most correlated with deviant innovation.

### Adjustment of Entrepreneur’s Psychological Capital to Deviant Innovation Behavior

**FIGURE 6:**
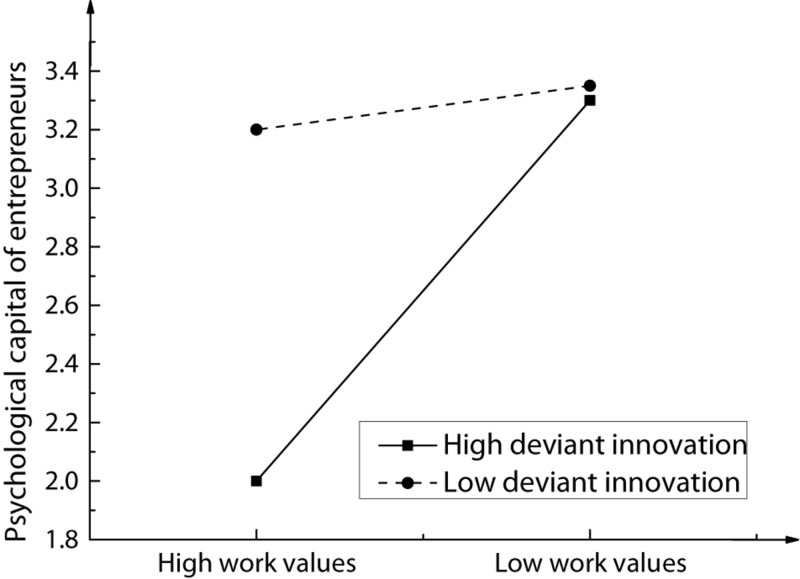
Adjustment of entrepreneur’s psychological capital to deviant innovation behavior.

**FIGURE 7:**
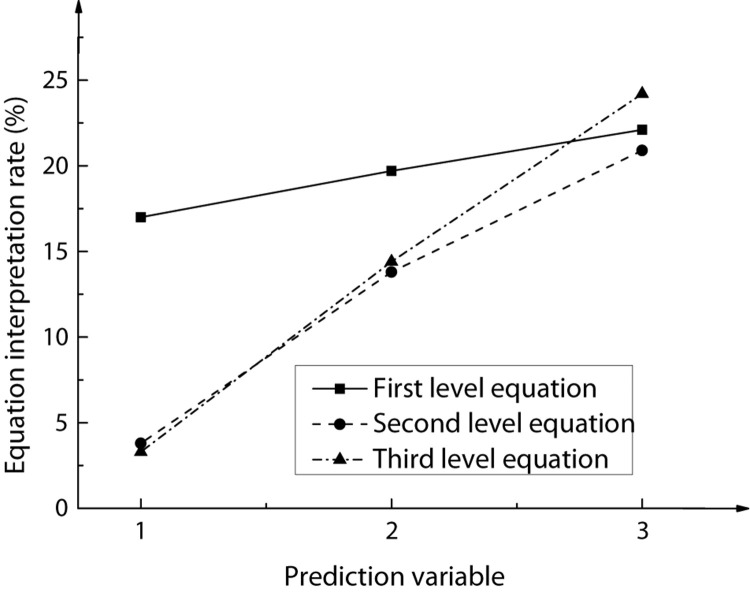
The change of interpretive rate of structural equation model before and after adjusting variables (1–3, respectively, represents the work values of entrepreneurs; work values of entrepreneurs + psychological capital of entrepreneurs; work values of entrepreneurs + psychological capital of entrepreneurs + deviant innovation).

**FIGURE 8:**
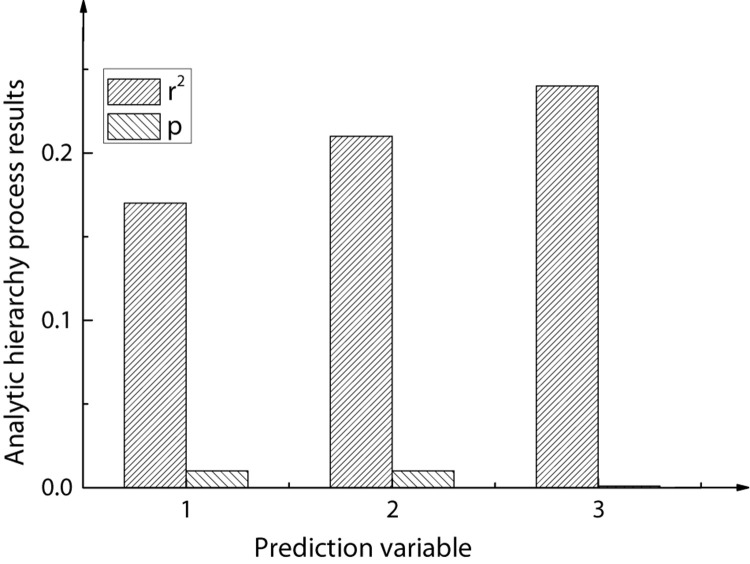
The change of AHP coefficient and significance level before and after introducing adjusting variables (the specific expression of 1–3 is the same as that in Figure 7).

Based on the above correlation analysis results of the selected variables, the entrepreneur’s work values were selected as independent variables, deviant innovation behavior as dependent variables, and entrepreneur’s psychological capital as adjusting variables. Through the AHP, the adjustment of entrepreneur’s psychological capital to the work values and deviant innovation behavior of the enterprise was discussed, and the corresponding regression results were analyzed, as shown in Figure 6. The structural equation model, before and after the entrepreneur’s psychological capital, was added as a regulating variable to explain the change of rate, as shown in Figure 7; the change of AHP coefficient *r*^2^ and significance level P is shown in Figure 8.

After the adjustment variable of entrepreneur psychological capital was added, the model explanation strength of the second-level equation increased significantly from 3.8 to 20.9% as compared with the first-level equation with an explanation rate of 17%, and the significance reached 0.001. The interaction term between entrepreneur work values and entrepreneur psychological capital was then introduced to the third-level equation. The final explanation rate of the equation for the relationship between variables was 24.2%, and the contribution rate of interaction terms to the equation was 3.3%, and the corresponding significance was 0.001. Therefore, it can be concluded that the psychological capital of entrepreneurs can regulate work values and deviant innovation behaviors of entrepreneurs.

## Discussion

In today’s environment of national innovation, enterprise innovation cannot be separated from the breakthrough of organization and individual members’ innovation. Entrepreneurs are a special group in an enterprise organization, and their work values and innovation concepts are very important to solve the innovation management problems of employees and even the whole organization (Hamdoun et al., 2018; Prado et al., 2018). Deviant innovation sometimes occurs among employees of enterprises and entrepreneurs. Psychological capital from the perspective of positive psychology has great potential in the promotion of organizational competitiveness and innovation breakthroughs (Kim et al., 2018). In this paper, considering the possibility of entrepreneurs’ psychological capital to promote the emergence and development of their deviant innovation behavior, as important members of an enterprise organization, individuals at different levels of psychological capital are likely to have different deviant innovation behavior when facing different management difficulties or other situations. The above results show that the psychological capital of entrepreneurs plays a regulatory role between their work values and deviant innovation behavior. Therefore, the psychological capital of entrepreneurs can promote the generation and development of deviant innovation behavior, which is of great significance to the improvement of enterprise innovation. Evidently, it is not difficult to find that positive psychology can play a positive role in innovation and creativity. It not only promotes the improvement of creativity but also has a certain regulatory role in the entire process of innovation and entrepreneurship.

Just as the guiding role of life values in people’s lives, work values play the same role in work. In the study of work values, demand orientation considers work values as the work characteristics pursued by the body in demand and activities. The standard judgment orientation holds that the work value has a guiding role in the work of individual employees and the selection of the conduct, which not only affects the individual’s self-orientation, but also impacts the objective requirements of the work (Li and Li, 2018; O’Connor and Kenter, 2019; Tomaževic et al., 2019). In the innovation activities of enterprises, entrepreneurs as an important group, greatly affect the innovation awareness of enterprises, in terms of their work values. The above research shows that they are important intermediary variables. The concept of entrepreneur’s work values was introduced into the research of deviant innovation behavior, and it was found that there was a significant correlation between entrepreneur’s work values and deviant innovation behavior. When the work values of entrepreneurs were taken as independent variables, the regulating effect of entrepreneurs’ psychological capital on their deviant innovation behavior was obvious (Wu et al., 2020).

The correlation among the psychological capital of entrepreneurs, the work values of entrepreneurs, the work remodeling of employees, and the deviant innovation behavior was analyzed, and it was found that the psychological capital of entrepreneurs plays a regulatory role in the deviant innovation behavior. To further develop the enterprise and make breakthroughs in innovation, the entrepreneurs who bear important responsibilities in the enterprise should focus on the encouragement of the innovation activities of employees in the enterprise, and promote their own and employees’ deviant innovation behavior to develop positively.

## Conclusion

Under the environment of social innovation, the generation and influence of deviant innovation was taken as the research object, the concept of psychological capital was introduced, and the work values of entrepreneurs and entrepreneurship work remodeling were added as variables. The measurement effect and correlation of each selected variable were tested and analyzed, and the role of entrepreneurs’ psychological capital in the regulation of deviant innovation behavior was explored. It was found that the reliability and validity of the measurement of each variable are satisfactory. Among the four variables, the psychological capital of entrepreneurs, the work values of entrepreneurs, and the deviant innovation behavior are highly correlated. The psychological capital of entrepreneurs can regulate their deviant innovation behavior. However, this paper only considers the work values of entrepreneurs as intermediate variables, and it was found that the psychological capital of entrepreneurs can regulate their deviant innovation behavior. In the future, more intermediate variables will be explored, and the relationship and influence between them will be further explored from other angles.

## Data Availability Statement

The raw data supporting the conclusions of this article will be made available by the authors, without undue reservation, to any qualified researcher.

## Ethics Statement

The studies involving human participants were reviewed and approved by the University Committee from Tongji University. The patients/participants provided their written informed consent to participate in this study.

## Author Contributions

WX contributed to writing of the manuscript. SZ contributed to the revision and validation. Both authors contributed to the article and approved the submitted version.

## Conflict of Interest

The authors declare that the research was conducted in the absence of any commercial or financial relationships that could be construed as a potential conflict of interest.
